# Spatial Normalization of ^18^F-Flutemetamol PET Images Using an Adaptive Principal-Component Template

**DOI:** 10.2967/jnumed.118.207811

**Published:** 2019-02

**Authors:** Johan Lilja, Antoine Leuzy, Konstantinos Chiotis, Irina Savitcheva, Jens Sörensen, Agneta Nordberg

**Affiliations:** 1 Department of Surgical Sciences, Nuclear Medicine, and PET, Uppsala University, Uppsala, Sweden; 2 Hermes Medical Solutions, Stockholm, Sweden; 3 Division of Clinical Geriatrics, Department of Neurobiology, Care Sciences, and Society, Karolinska Institutet, Huddinge, Sweden; 4 Department of Nuclear Medicine, Karolinska University Hospital, Huddinge, Sweden; and; 5 Theme Aging, Karolinska University Hospital, Huddinge, Sweden

**Keywords:** Alzheimer disease, amyloid-β, PET, ^18^F-flutemetamol, adaptive template

## Abstract

Though currently approved for visual assessment only, there is evidence to suggest that quantification of amyloid-β (Aβ) PET images may reduce interreader variability and aid in the monitoring of treatment effects in clinical trials. Quantification typically involves a regional atlas in standard space, requiring PET images to be spatially normalized. Different uptake patterns in Aβ-positive and Aβ-negative subjects, however, make spatial normalization challenging. In this study, we proposed a method to spatially normalize ^18^F-flutemetamol images using a synthetic template based on principal-component images to overcome these challenges. **Methods:** ^18^F-flutemetamol PET and corresponding MR images from a phase II trial (*n* = 70), including subjects ranging from Aβ-negative to Aβ-positive, were spatially normalized to standard space using an MR-driven registration method (SPM12). ^18^F-flutemetamol images were then intensity-normalized using the pons as a reference region. Principal-component images were calculated from the intensity-normalized images. A linear combination of the first 2 principal-component images was then used to model a synthetic template spanning the whole range from Aβ-negative to Aβ-positive. The synthetic template was then incorporated into our registration method, by which the optimal template was calculated as part of the registration process, providing a PET-only–driven registration method. Evaluation of the method was done in 2 steps. First, coregistered gray matter masks generated using SPM12 were spatially normalized using the PET- and MR-driven methods, respectively. The spatially normalized gray matter masks were then visually inspected and quantified. Second, to quantitatively compare the 2 registration methods, additional data from an ongoing study were spatially normalized using both methods, with correlation analysis done on the resulting cortical SUV ratios. **Results:** All scans were successfully spatially normalized using the proposed method with no manual adjustments performed. Both visual and quantitative comparison between the PET- and MR-driven methods showed high agreement in cortical regions. ^18^F-flutemetamol quantification showed strong agreement between the SUV ratios for the PET- and MR-driven methods (*R*^2^ = 0.996; pons reference region). **Conclusion:** The principal-component template registration method allows for robust and accurate registration of ^18^F-flutemetamol images to a standardized template space, without the need for an MR image.

After the first successful study using the ^11^C-labeled amyloid-β (Aβ)–selective ligand Pittsburgh compound B (1), Aβ imaging with PET has exerted a rapid and extensive influence on Alzheimer disease research. As a result of the short half-life of the ^11^C-radioisotope, however, an onsite cyclotron and specialized radiochemistry infrastructure are required, limiting the utility of ^11^C-Pittsburgh compound B as a clinical diagnostic tool. As such, several Aβ-specific PET tracers radiolabeled with the longer-lived ^18^F-radioisotope have been developed for clinical applications. To date, 3 such compounds have been approved by the Food and Drug Administration and the European Medicines Agency, including ^18^F-florbetapir (2,3), ^18^F-florbetaben (4,5), and ^18^F-flutemetamol (6,7).

Though currently validated for visual assessment only, in which scans are classified as negative (normal) or positive (abnormal) by a trained reader, there is evidence to suggest that the incorporation of quantitative approaches for use with currently approved Aβ PET tracers may reduce interreader variability (8) and aid in monitoring treatment effects from anti-Aβ drugs (9). Quantification typically involves computation of an SUV ratio (SUVR), in which late-duration tracer uptake within target regions is normalized to that within a reference tissue such as the cerebellum or pons (1,3,4,7). As a requisite for SUVR computation, a PET image must first be parcellated into anatomically meaningful regions; the gold standard for this type of approach requires access to a subject’s T1-weighted MR image and manual delineation of volumes of interest (VOIs) in native space. This method, however, is time-consuming and may be subject to interreader variability; further, structural imaging can prove challenging to perform in clinical settings. As such, PET-only approaches have been developed by which, after normalization to a reference space, division of the Aβ image into VOIs and subsequent calculation of SUVR is achieved using a computer-generated, predefined regional atlas.

One of the challenges inherent in current ^18^F-Aβ PET tracers is their capacity for high uptake in both gray and white matter. Though increased cortical uptake occurs in proportion to fibrillary Aβ levels, nonspecific white matter uptake is characteristically seen regardless of fibrillary Aβ load (Fig. 1). The different patterns of uptake across Aβ-positive (Aβ+) and -negative (Aβ−) images can therefore result in a systematic bias when a standard, single-template, PET-driven registration method is used. Though use of a subject’s MR image stands as a possible solution to this challenge, MRI is not always available as part of routine clinical workup, highlighting the relevance of a PET-based method able to resolve the bias imposed by variability in Aβ ligand uptake.

**FIGURE 1.**
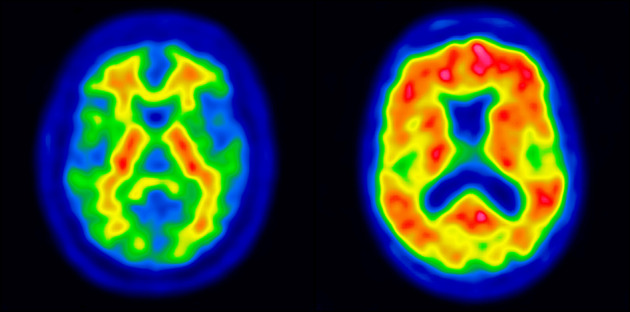
Typical patterns of Aβ− (left) and Aβ+ (right) subjects.

To overcome these problems, we developed a fully automated PET-only registration method using a synthetic template based on principal-component images. In this approach, the linear combination of the first and second components from a principal-decomposition analysis of ^18^F-flutemetamol images was used to model a synthetic template spanning the whole range from Aβ− to Aβ+. The synthetic template was then used to drive registration to standard space. Here, we describe the creation of the principal-component template model, its integration into an image registration algorithm, and the validation of our method against an MR-driven approach for spatial normalization (SPM12).

## MATERIALS AND METHODS

### Subjects and Imaging

The study population consisted of 117 subjects divided across 2 cohorts: a template creation cohort and a registration validation cohort (Table 1). The template creation cohort consisted of 70 subjects (25 cognitively normal healthy volunteers, 19 amnestic patients with mild cognitive impairment, and 26 Alzheimer disease patients) from a ^18^F-flutemetamol phase II study (6) and was approved by the Ethical Committees of the participating sites. The registration validation cohort consisted of 47 patients (14 with mild cognitive impairment, 27 with Alzheimer disease, 5 with non-Alzheimer disease, and one with dementia not otherwise specified) from an ongoing study at the Karolinska University Hospital, Huddinge, Sweden, investigating the clinical utility of ^18^F-flutemetamol PET in patients with an unclear diagnosis. The Regional Human Ethics Committee of Stockholm, Sweden, and the Isotope Committee of Karolinska University Hospital Huddinge approved this study. For both studies, all patients gave written informed consent.

**TABLE 1:** Demographic and Clinical Information for Template Creation and Validation Cohorts

				Sex (*n*)		
Cohort	Group	*n*	Age (y)	M	F	MMSE
Template creation	Healthy volunteers	25	58 (44, 72)	12	13	—
	Amnestic mild cognitive impairment	19	71 (69, 80)	1	9	27–30
	Alzheimer disease	26	72 (64.3, 74)	12	14	15–26
Registration validation	Mild cognitive impairment	14	60 (56, 64)	5	9	24–29
	Alzheimer disease	27	66.5 (63.3, 73)	9	18	23–26
	Non-Alzheimer disease*	5	63 (62, 66)	2	3	24–27
	Dementia, not otherwise specified	1	63	1	0	22

* 3 cases of vascular dementia and 2 of frontotemporal dementia.

For age, data are median followed by first and third quartiles in parentheses; for MMSE, data are range.

Data from the template creation cohort were used to create the principal-component templates and to evaluate the image registration method, whereas data from the registration validation cohort were used to validate the spatial normalization. For the template creation cohort, the ^18^F-flutemetamol PET imaging protocol consisted of six 5-min frames 85 min after injection of approximately 180 MBq, with scanning performed at 3 different centers using 3 different scanners (Biograph PET/CT and ECAT EXACT HR+ [Siemens] and Advance [GE Healthcare]); structural T1-weighted MRI data were acquired for all subjects. For the registration validation cohort, the ^18^F-flutemetamol protocol consisted of a 20-min (list-mode) scan 90 min after injection of approximately 185 MBq (Biograph mCT PET/CT; Siemens). Structural T1-weighted MRI data were acquired at several radiology departments in Stockholm and neighboring counties, with different platforms and protocols.

### Reference Space and Anatomic Regions

**FIGURE 2.**
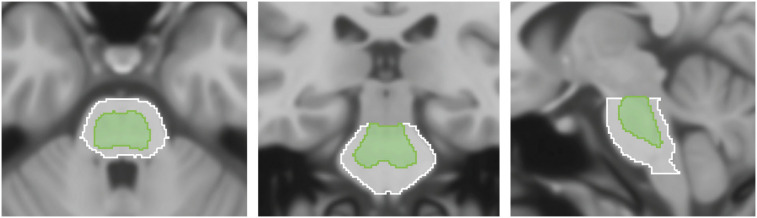
Centiloid pons (white) and modified version of centiloid pons (ThPons, green), based on voxels having highest ^18^F-flutemetamol uptake.

Montreal Neurological Institute space was used as a reference space, together with the 1-mm isotropic T1-weighted MR template from the International Consortium of Brain Mapping (10). The following VOIs were adapted from the Centiloid project (11), a recently proposed method aiming to facilitate comparison and combination of Aβ PET data through the use of a linear scaling procedure: cerebellar gray (CG), whole cerebellum (WC), WC plus brain stem, pons, and global cortical target (CTX). Included in the CTX meta-VOI were brain regions known to typically display a high load of amyloid, including the frontal, temporal, and parietal cortices, as well as the precuneus, anterior striatum, and insular cortex (11). In addition, we added a fifth VOI by modifying the Centiloid pons VOI through the selection of only those voxels containing high ^18^F-flutemetamol uptake (Fig. 2). This threshold version of the Centiloid pons is referred to as ThPons.

### Principal-Component Template and Spatial Normalization

Principal-component analysis is a statistical technique to investigate the variance–covariance structure of a set of variables. In general, principal-component analysis serves 2 purposes: data reduction and interpretation. When one is working with highly dimensional data, principal-component analysis may be a good way of reducing the dimensionality of data, thereby revealing relationships that would otherwise be hard to find.

Given *n* images with *p = rows × cols × slices* voxels, a matrix **X**^*p×n*^ can be formed. The (unbiased) sample variance–covariance matrix, **C**^*n×n*^, of **X** can then be calculated as

$$C=\frac{D'D}{n-1},$$

where **D**^*p×n*^ = (**X**^*p×n*^ − **μ**^*P×1*^**1**^*1×n*^), where **1**^*1×n*^ is a row vector of ones and **μ** is simply the mean image where voxel *i* (*i* = 1,…,*p*) is calculated as

$$\mu_{i}=\frac{1}{n}\sum_{i=1}^{n}x_{i}.$$

The voxel-based principal components can then be calculated using singular value decomposition,

C=VΛV',

where **V**^*n×n*^ is the eigenvector matrix whose *i*^th^ (*i* = 1,…,*n*) column is the eigenvector, **q**_**i**_, **Λ**^*n×n*^ is the diagonal matrix whose diagonal elements, λ_*i*_, are the corresponding eigenvalues, and **V**′ is the transpose of **V**. A principal-component image, *I*_*PCi*_, is then calculated by multiplying **D** by one of its eigenvectors, **q**_**i**_:

$$I_{\text{PC}i}=D\times q_{i}.$$

**FIGURE 3.**
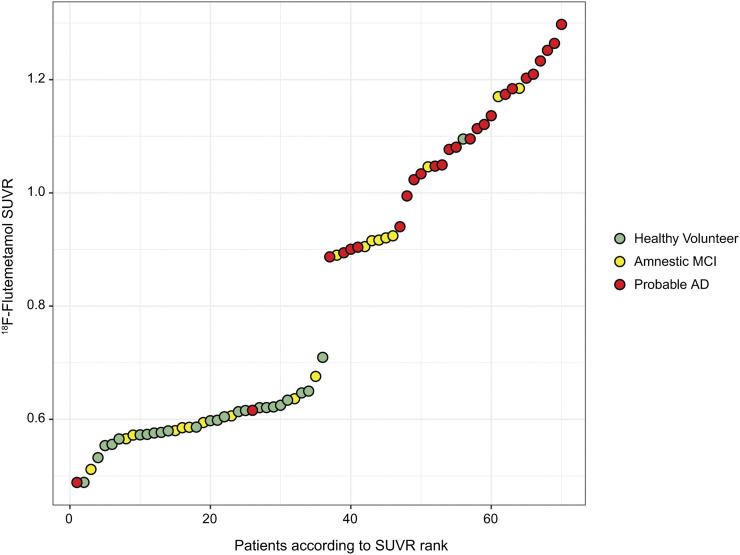
^18^F-flutemetamol CTX SUVR (using pons as reference region) on *y*-axis (template creation cohort) against patients, ranked according to SUVR, on *x*-axis.

First, all MR and corresponding ^18^F-flutemetamol images were coregistered. We then spatially normalized all images to Montreal Neurological Institute space using the MR-driven registration provided by SPM12. Images were then intensity-normalized using the pons region. To make sure the images in the template creation cohort spanned the whole range from Aβ− to Aβ+, we calculated the cortical SUVRs using CTX as target region and pons as reference region (Fig. 3). SUVR findings for the registration validation cohort are shown in Supplemental Figure 1 (supplemental materials are available at http://jnm.snmjournals.org).

**FIGURE 4.**
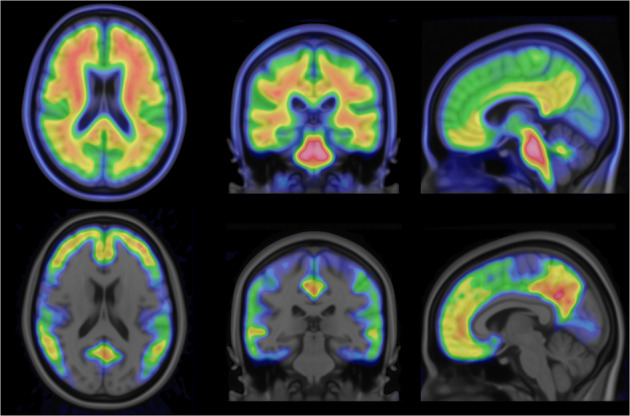
From left to right: axial, sagittal, and coronal views of principal-component image 1 (top) and principal-component image 2 (bottom).

Principal-component images for all 70 spatially and intensity-normalized ^18^F-flutemetamol images in the template creation cohort were calculated. Principal-component images were then sorted on the basis of their eigenvalues to determine the order of significance for each component. We decided to use the first 2 principal components, explaining 94.6% of the variance, to generate a synthetic template. Any number of components could be used, but since the remaining 68, higher-order, components describe an accumulated variance of only 5.4% of the total variance, they were considered not to contribute much relevant information on image registration. Figure 4 shows transaxial, coronal, and sagittal views of the first principal-component image (*I*_PC1_) and the second principal-component image (*I*_PC2_) using the recommended rainbow color scale (12).

A synthetic template image, *I*_*synthetic*_, could now be modeled by a linear combination of *I*_PC1_ and *I*_PC2_, where a weight, *w*, ranging from −1.0 to 1.0 is multiplied by *I*_PC2_ according to:

$$I_{synthetic}=\;I_{\text{PC}1}+wI_{\text{PC}2}$$

**FIGURE 5.**
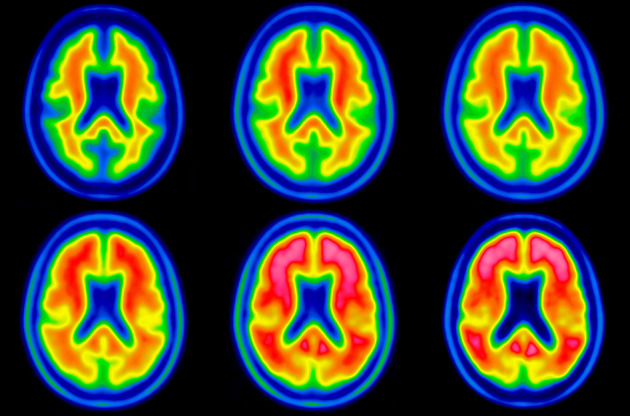
Synthetic template images showing characteristic ^18^F-flutemetamol uptake pattern going from most negative case (upper left) to most positive case (lower right). Value of weight ranges from −1.0 (upper left) to 1.0 (lower right) and is increased by 0.4 going from left to right, top to bottom.

A value of −1.0 for weight *w* will correspond to an Aβ− subject, whereas a value of 1.0 will correspond to an Aβ+ subject. Figure 5 shows the range of synthetic template images across the range (−1.0 to 1.0) of possible *w* values.

A registration method was developed allowing the weight *w* to be incorporated into the optimization method together with the parameters for spatial transformation as described by Lundqvist at al. (13). This development allows the registration method to iteratively find the best set of spatial transformation parameters for a patient’s ^18^F-flutemetamol scan to fit the optimal template for this particular scan. As an initial step, a multiresolution global 12-parameter affine registration is used. When the global registration has converged, a brain mask without ventricles is used to refine the registration of the cortical areas of the brain by continuing to improve the affine transform, as well as by adding a higher-order deformation in the form of a second-order polynomial transformation (14). Once converged, the registration of the brain stem and cerebellum is refined. For the first 2 steps, normalized mutual information was used as a similarity metric, whereas for the last step, normalized cross correlation was used. Powell’s algorithm (15) was used for optimization throughout the whole registration.

### Refined Registration of Pons and Cerebellum

**FIGURE 6.**
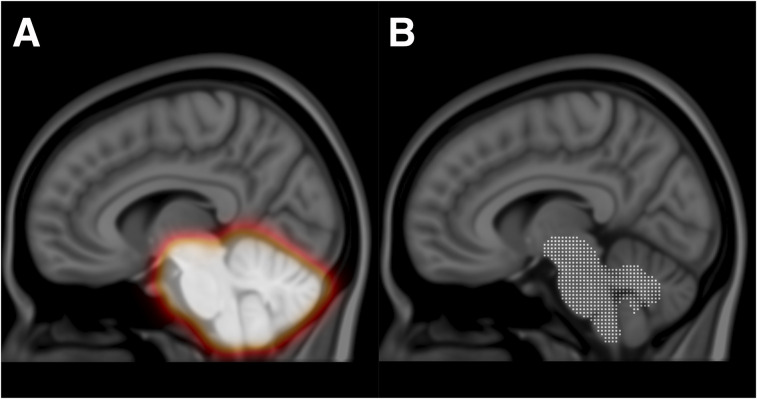
Cerebellum and brain stem mask (A) and subsampling of high ^18^F-flutemetamol uptake of cerebellum and brain stem mask (B).

The quality of the registration of the reference region is important for quantification of Aβ images. Because of the low anatomic information in Aβ PET images, a registration method with a high number of degrees of freedoms may not be feasible. The constraints of the second-order polynomial deformation field may, on the other hand, not be sufficient to get a good local registration of the reference regions. Therefore, we added a final registration step to the registration algorithm by which we allow for a 6-parameter rigid registration of the pons and cerebellum. A volumetric binary mask covering the whole cerebellum and brain stem was created. The mask was then smoothed using a 3-dimensional gaussian filter (Fig. 6A). When the reference region was registered, not all voxels within the mask were used; rather, it was subsampled by thresholding *I*_*synthetic*_ using only the characteristic high-uptake voxels to drive the registration. The threshold was determined by calculating an intensity histogram for all voxels of *I*_*synthetic*_ within the cerebellum and brain stem mask and then calculating the voxel value at 85% of the intensity histogram (Fig. 6B). The calculated transform was then applied using to the whole cerebellum and brain stem mask. To avoid discontinuities in the final transformed image, the smoothed mask was used as a weight for the calculated transform, by which values completely outside the mask are not affected and values within the mask are affected in the order of the value in the smoothed mask, ranging from 0.0 to 1.0, with a value of 1.0 meaning that the calculated transform is fully applied.

### Experiments

Coregistered ^18^F-flutemetamol images were spatially normalized to the Montreal Neurological Institute T1 template in 2 ways: first, using transforms derived by MR-driven registration as provided by SPM12 (http://www.fil.ion.ucl.ac.uk/spm), and second, using the principal-component template registration method.

### Quality of Spatial Normalization

**FIGURE 7.**
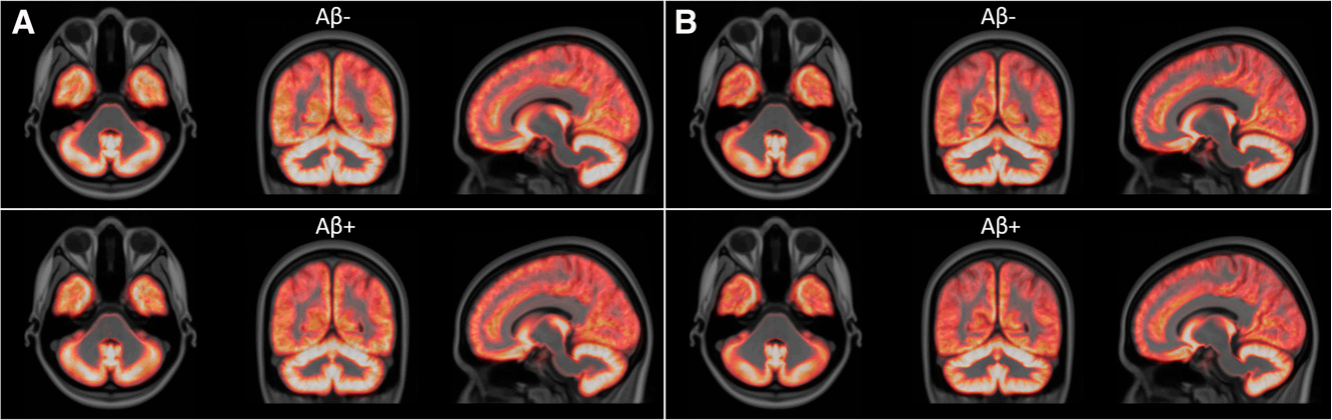
Average gray matter probabilistic maps (template creation cohort) for Aβ− and Aβ+ subjects using principal-component template registration (A) and SPM12 registration (B).

Using spatially normalized MR images from the template creation cohort, we created gray and white matter probabilistic tissue masks for both registration methods. The amount of gray matter and white matter in CG and CTX VOIs was based on the probabilistic tissue maps. Paired *t* tests using a commercial software package (Matlab, R2016a; The MathWorks Inc) were performed to evaluate differences in the amount of gray matter and white matter in CG and CTX VOIs between the 2 registration methods. Mean gray matter images for Aβ− and Aβ+ images were then created for each registration method (Fig. 7), with images then visually inspected for systematic differences in registration quality tied to Aβ status (negative or positive).

### Quantitative Comparison with MR-Driven Registration

For both the template creation and the validation cohorts, cortical SUVRs were calculated using all reference regions and both registration methods. The correlation between MR-driven and principal-component template registration methods was then calculated separately for each cohort.

## RESULTS

### Quality of Spatial Normalization

All scans were successfully spatially normalized, with no manual adjustments performed. The amount of gray matter in the CG VOI was higher for the principal-component template approach (*P* < 0.001), whereas the amount of white matter was lower (*P* < 0.001) (Supplemental Figs. 2A and B). Similar results were found when the CTX VOI was used (*P* < 0.001) (Supplemental Figs. 2C and D). For both regions, the principal-component template registration method yielded lower variance in the results in the amount of gray matter as well as for the results in the amount of white matter.

Visual inspection of the mean gray matter images created from the SPM12 registered MR images did not show any apparent systematic differences between Aβ− and Aβ+, nor did the mean gray matter images created using the principal-component template registration method. Further, we could not see any apparent differences between the 2 registration methods based on the visual inspection of the gray matter mean images for Aβ− images, nor for Aβ+ images.

### Quantitative Comparison with MR-Driven Registration

Comparison of quantification results using ^18^F-flutemetamol–driven principal-component template registration and MR-driven SPM12 registration showed good agreement (Table 2).

**TABLE 2:** Correlation Between SUVRs for CTX Region Computed with Principal-Component Template Registration and SPM12

Reference region	Cohort	*R* ^2^
CG	Template creation	0.978
	Registration validation	0.984
WC	Template creation	0.991
	Registration validation	0.992
WC + brain stem	Template creation	0.993
	Registration validation	0.993
Pons	Template creation	0.993
	Registration validation	0.986 (0.993*)
ThPons	Template creation	0.995
	Registration validation	0.996

* Results after removal of 2 subjects for whom visual assessment showed that principal-component–based registration was superior to SPM12 registration, based on fit to centiloid pons.

For the registration validation cohort, the coefficient of determination, *R*^2^, between SUVRs for the CTX region computed with both methods ranged from 0.984 with CG as reference region to 0.996 with ThPons as reference region (Supplemental Fig. 3).

By visual inspection, it was noted that 2 subjects, A and B, in the registration validation cohort had a better fit to pons using the principal-component registration than using the SPM12 registration (Supplemental Fig. 4). Removing these 2 subjects from the analysis gave an *R*^2^ value of 0.993 using pons as reference region.

## DISCUSSION

Quantification of tracer uptake has the potential to aid clinicians in interpreting Aβ PET images, as a complement to visual assessment. This approach can increase clinical confidence, particularly when the images are difficult to interpret. For this approach, regions of interest and reference regions need to be defined to provide the image interpreter with a quantitative measure unaffected by parameters such as the administered dose or patient body composition. Commonly, these regions are defined through spatial normalization of the images to a template space, in which a predefined atlas can be applied to estimate SUVR. However, the bimodal appearance of Aβ imaging (16) presents a well-known obstacle to the use of single-modality spatial normalization of PET images to a template space, since the use of a common PET template image for both positive and negative images commonly leads to suboptimal registration because of the completely different image characteristics.

We have here demonstrated the implementation and creation of a synthetic Aβ template using principal-component decomposition of a set of Aβ scans ranging from Aβ− to Aβ+, registered to Montreal Neurological Institute space. Even though it would be possible to use a larger number of components, the synthetic template was created using a linear combination of the first 2 principal components, excluding the higher-order components that contributed minimally to image appearance. The second-order component was identified as being responsible for the signal related to specific binding, thus representing the difference between positive and negative images. As seen in Figure 4, this component accurately identifies cortical regions associated with Aβ binding, excluding the motor, visual, and cerebellar cortices. In our template creation procedure, the weight of this component is determined iteratively during the image registration procedure, resulting in a template with an optimal likeness to the positive–negative range from the patient data being normalized. Another advantage is that the registration to the template of the patient image is performed using a template with image characteristics similar to the patient image, improving registration quality.

The proposed method focuses on the accuracy of the registration of cortical and reference regions. Using a global deformation field, as provided by a second-order polynomial transform that has relatively few degrees of freedom, it is possible to compensate for global differences in anatomy. However, we noticed that the size of the ventricles varied across subjects; therefore, as a precaution against any potential bias in the registration, we did not include the ventricles in the final step of the global registration, limiting the performance of the registration around the ventricles.

Although compensating for global differences in anatomy, the global deformation field may not be able to compensate for differences in the reference regions. We therefore introduced a final registration step allowing for refinement of the registration of the reference regions. An interesting finding was the quality of the registration of the reference regions in subjects A and B, for whom we believe the proposed method showed a more accurate registration than the reference method. This greater accuracy may be due to the choice of reference method, with the MR-driven registration provided by SPM12 allowing for only a global deformation field using a linear combination of low-frequency basis functions.

Diagnosing pathologic Aβ binding using PET at the individual patient level was recently shown to have a significant impact on diagnostic confidence and drug treatment (17–19). The PET-based diagnosis is currently a dichotomous process performed using visual criteria. Cases with borderline changes are often difficult to classify visually in a clinical routine setting, especially for readers without extensive experience. Further, disease-modifying drugs targeting Aβ plaques may have only modest effects on brain levels, resulting in changes to the Aβ PET signal that are not visually apparent. Automated quantification using the proposed method might increase reader certainty and further the clinical adoption of Aβ imaging, including within the context of clinical trials. Moreover, since the method proposed here appears to be at least as accurate as the dual-scan concept used by the gold standard SPM12, the new method might simplify such studies by obviating a separate MRI scan. Further, because of the choice of data for the generation of the template and for validation, the method may be considered insensitive to reconstruction method and scanner type.

Other methods using adaptive templates (13) and principal-component–derived templates (20) have been proposed for ^11^C-Pittsburgh compound B and ^18^F-flutemetamol, respectively. In the study by Fripp et al. (20), although their method, similar to ours, used a principal-component approach to generate an adaptive template, spline-based transformations were used for the normalization step; as a result, their approach carries a heavy computational cost (>6 h). By comparison, an average processing time of 20 s is required to process a subject using our method. Further, our work extends that of Fripp et al., showing that a principal-component–driven template approach can be successfully implemented with ^18^F-flutemetamol and, possibly, additional approved ^18^F-Aβ tracers. In the study by Lundqvist et al. (13), linear regression was used to generated intercept (fixed) and slope images; the latter, in combination with a weighting factor, was then used to generate a template. Though their regression-derived slope image provides a measure similar to our second principal-component image, visual comparison of the resulting templates suggests greater accuracy with our proposed method. Moreover, in contrast to their method, for which a patent is pending (21), our approach is unpatented, potentially facilitating collaborative projects aiming to validate our template using additional Aβ tracers.

## CONCLUSION

The proposed method allows for robust and accurate registration of ^18^F-flutemetamol images to template space, without additional imaging procedures. This advantage might simplify the clinical use of quantification in Aβ PET imaging. The use of adaptive templates—a promising strategy—might be expanded to other tracers with potential clinical applications, such as those for tau or dopamine synthesis and transport.

## DISCLOSURE

The ^18^F-flutemetamol phase II study was designed and sponsored by GE Healthcare. ^18^F-flutemetamol data collected in the validation cohort were largely sponsored by Vinnova—the Swedish Governmental Agency for Innovation Systems, grant 2013-05175. Johan Lilja is a former employee of GE Healthcare and currently holds a position at Hermes Medical Solutions. No other potential conflict of interest relevant to this article was reported.
